# Dynamic of the human gut microbiome under infectious diarrhea

**DOI:** 10.1016/j.mib.2022.01.006

**Published:** 2022-04

**Authors:** Hao Chung The, Son-Nam H Le

**Affiliations:** 1 Oxford University Clinical Research Unit, Ho Chi Minh City, Viet Nam; 2 School of Biotechnology, International University, Vietnam National University, Ho Chi Minh City, Viet Nam

## Abstract

Despite the widespread implementation of sanitation, immunization and appropriate treatment, infectious diarrheal diseases still inflict a great health burden to children living in low resource settings. Conventional microbiology research in diarrhea have focused on the pathogen’s biology and pathogenesis, but initial enteric infections could trigger subsequent perturbations in the gut microbiome, leading to short-term or long-term health effects. Conversely, such pre-existing perturbations could render children more vulnerable to enteropathogen colonization and diarrhea. Recent advances in DNA sequencing and bioinformatic analyses have been integrated in well-designed clinical and epidemiological studies, which allow us to track how the gut microbiome changes from disease onset to recovery. Here, we aim to summarize the current understanding on the diarrheal gut microbiome, stratified into different disease stages. Furthermore, we discuss how such perturbations could have impacts beyond an acute diarrhea episode, specifically on the child’s nutritional status and the facilitation of antimicrobial resistance.

## Highlights

• Infectious diarrhea disrupts the gut microbiome and reduce its diversity.
• Enterobacteriaceae, *Streptococcus* and oral bacteria bloom in gut following diarrhea.
• Mucin-degrading *Bacteroides* is keystone species for microbiome recovery.
• Diarrhea-induced dysbiosis has impacts on malnutrition and horizontal gene transfer.

**Current Opinion in Microbiology** 2022, **66**:79–85

This review comes from a themed issue on **Microbiota**

Edited by **Lindsay Hall and Melanie Schirmer**

For complete overview of the section, please refer to the article collection, “Microbiota”

Available online 1st February 2022

**https://doi.org/10.1016/j.mib.2022.01.006**

1369-5274/© 2022 The Authors. Published by Elsevier Ltd. This is an open access article under the CC BY license (http://creativecommons.org/licenses/by/4.0/).

## Introduction

Infectious diarrhea remains a major global health problem. It is characterized by the passage of at least 3 loose, liquid stool per day in patients, and is caused by a wide array of etiologies, including viruses (rotavirus, norovirus), bacteria (*Campylobacter*, *Salmonella*, *Shigella*, *Vibrio*, *Escherichia coli*), and parasites (*Cryptosporidium*, *Entamoeba*, *Giardia*) [1, 2, 3, 4]. Approximately 1.3 million deaths yearly are attributed to diarrhea, of which ∼500 000 target children under five years-old, making diarrhea the fourth leading cause of child mortality [4]. This burden disproportionately affect children in low and middle income countries (LMICs), where ∼950 million diarrhea cases occur annually against a backdrop of poor access to sanitation, good nutrition and healthcare [4].

The microbial communities inhabiting the gastrointestinal tract is an integral component to human health. The density and diversity are greatest for communities in the colon (termed the gut microbiota), which mainly consist of anaerobic bacteria of phyla Bacteroidetes and Firmicutes [5]. Perturbation in the gut microbiota and its encompassing environment (gut microbiome dysbiosis) has been linked to health conditions as varied as cancer [6], metabolic diseases [7], and depression [8], which are mostly chronic and non-communicable. Diarrhea presents a major dysbiosis event, as increased bowel movements and fluid secretion destabilize the gut environment. The acute nature of diarrhea also requires longitudinal observations to fully capture the rapid microbiome changes. Though most diarrhea episodes are self-limiting, patients are frequently treated with oral rehydration, zinc supplement, probiotics and antimicrobials (in case of dysentery or bacterial infections) [9,10]. The latter two treatments further introduce destabilizing effects. Additionally, the gut microbiome composition and succession in young children are highly varied and dynamic, depending on geography, birth term, mode of delivery, breastfeeding, time of weaning, and nutritional status [11, 12, 13]. All these factors combined complicate interpretations from diarrhea microbiome studies. In the scope of this review, we summarize the current understanding on the impact of infectious diarrhea on gut microbiome, focusing on its dynamic in different disease phases, as well as the effect of such dysbiosis beyond acute diarrhea. Similar to other disciplines, most diarrhea microbiome research rely on the culture-independent approach to offer a thorough representation of the microbial community, accessed via 16S rRNA amplicon or shotgun metagenomic sequencing of fecal samples. We searched the PubMed database using the keywords ‘((microbiota[MeSH Terms]) OR (microbiome[MeSH Terms])) AND (diarrhea[MeSH Terms])’, and included articles which mentioned the investigation of the gut microbiome in infectious diarrhea.

## Dysbiosis in the early phase of diarrhea

**Figure 1:**
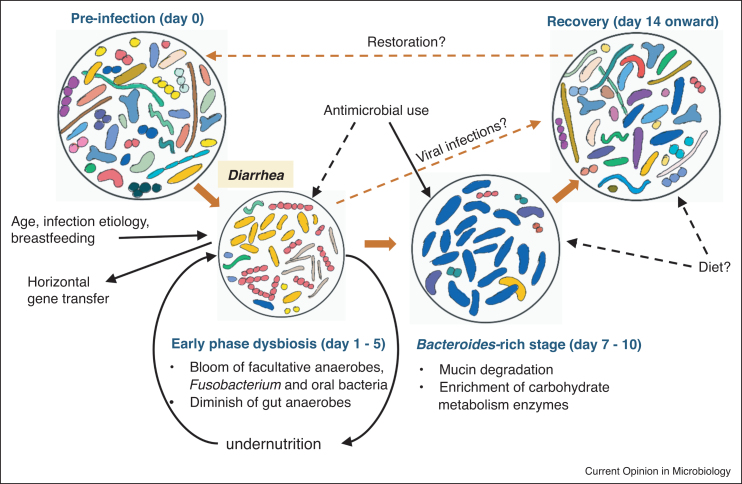
Schematic representation of gut microbiome succession under infectious diarrhea. Each circle depicts a microbiome state, with alpha-diversity proportional to the circle’s size and richness illustrated by bacteria of different shapes and colors. Solid brown arrows delineate the directional transitions between microbiome states, while dashed brown arrows represent probable transitions under the annotated conditions. Solid black arrows denote factors which impact the configuration of the microbiome state (or vice versa), and dashed black arrows indicate such probable/uncertain impacts.

We define the disease’s early phase as the period when diarrhea symptoms have not subsided, frequently within the first three to five days since disease onset or presentation to hospitals. Diarrhea brings forth a marked reduction in taxonomic richness and diversity, compared to age-matched and location-matched healthy individuals [14^•^,15^•^,16^••^]. Repeated washouts could greatly erode the microbiota, and higher water content in diarrheal stool (lower bowel transit time) has been associated with lower alpha-diversity, as observed previously in European adults [17]. The gut microbiome undergoes a dramatic taxonomic change upon diarrhea’s onset, favoring the proliferation of fast-growing facultative anaerobes. Proteobacteria (mostly Enterobacteriaceae/*E. coli*) and *Streptococcus* (mainly *Streptococcus salivarius* and *Streptococcus gallolyticus*) are most significantly enriched during this early phase, and could account for up to 80% in relative abundance in the fecal microbiomes [14^•^,15^•^,18^••^,^19^, ^20^, ^21^] (Table 1, Figure 1). The bloom of these bacteria is facilitated by the transiently oxygenated gut environment during diarrhea, evidenced by the respective elevation in genes encoding low-affinity cytochrome oxidases [18^••^]. This increased abundance is coupled with a drastic disappearance of obligate anaerobic gut commensals (*Blautia*, *Prevotella*, *Faecalibacterium*, Lachnospiraceae, Ruminococcaceae, etc.) [14^•^,15^•^], leading to a depletion of associated metabolites such as short chain fatty acid (SCFAs) [22,23]. Diarrheagenic bacteria, however, are usually of transient and/or low abundance (except for *Vibrio cholerae* in the first day) [15^•^,18^^•^^**^^•^^**,^24^]. Nevertheless, such global dysbiosis was not observed in all patients with diarrhea, and a portion of infected patients retain fecal microbiomes highly resembling those found in healthy controls [15^•^,^24^]. Particularly, the gut microbiome of children with diarrhea could be grouped into four enterotypes, each predominated by a taxon: *Bifidobacterium*, *Bacteroides*, *Streptococcus*, or *Escherichia*. Younger age (<20 months-old) and exclusive breastfeeding were associated with the *Bifidobacterium* enterotype, while poor nutritional status and older age were linked to the *Escherichia* enterotype [15^•^]. It is inconclusive how these different initial configurations affect clinical outcome and recovery, but higher relative abundance of *Streptococcus* has shown positive correlation with hospitalization length or diarrhea duration [25,26].

**Table 1:** Summary of diarrhea microbiome studies, by order of appearance in this review. ND: Not determined; NA: not available

Reference	Study location	No. diarrhea patients	Length of follow-up	Study method	Diarrhea etiologies	Antibiotic treatment	Taxa abundant in diarrhea dysbiosis	Taxa depleted in diarrhea dysbiosis
Pop *et al.* [14^•^]	Gambia, Mali, Kenya, Bangladesh	508	–	16S rRNA	Multiple (ND)	NA	*Escherichia*, *Granulicatella*, *Streptococcus*	*Prevotella*, *Bacteroides*, *Megasphaera*
Chung The *et al.* [15^•^]	Vietnam	145	–	16S rRNA	*Salmonella*, *Shigella*, *Campylobacter*, norovirus, rotavirus	No	*Streptococcus*, *Escherichia*, *Fusobacterium*, oral bacteria	Clostridiales, Erysipelotrichales
David *et al.* [18^••^]	Bangladesh	41	1–6 months	Shotgun metage-nomic	*Vibrio cholerae*, *Escherichia coli*	Azithromycin	*Escherichia*, *Enterococcus*, *Streptococcus*	*Bacteroides*, *Prevotella*, *Roseburia*
Hsiao *et al.* [19]	Bangladesh	7	3 months	16S rRNA	*Vibrio cholerae*	Azithromycin	*Streptococcus*, *Fusobacterium*, *Granulicatella*, *Escherichia*	*Bacteroides*, *Prevotella*, *Blautia*, *Faecalibacterium*
Monira *et al.* [20]	Bangladesh	9	1 month	16S rRNA	*Vibrio cholerae*	Erythromycin	Enterobacteriaceae	Bacteroidaceae, Bifidobacteriaceae, Ruminococcaecea
Sohail *et al.* [21]	Qatar	39	–	16S rRNA	Rotavirus	NA	Proteobacteria, Fusobacteria, *Streptococcus*	*Bacteroides*, Firmicutes
Singh *et al.* [24]	USA	200	1−14 weeks	16S rRNA	*Campylobacter*, *Salmonella*, *Shigella*, *E. coli*	NA	Enterobacteriaceae, Pasteurellaceae, Lactobacillales,	*Bacteroides*, *Prevotella*, *Roseburia*, *Blautia*
Becker-Dreps *et al.* [27]	Nicaragua	25	2 months	16S rRNA	*E. coli*, *Shigella*, norovirus, parasites	Yes	*Fusobacterium*, *Cetobacterium*, *Achromobacter*	*Lactobacillus*, Clostridiales
Gallardo *et al.* [34]	Chile	63	–	16S rRNA	*Escherichia coli*, viruses	No	Enterobacteriaceae, *Pseudocitrobacter*, *Enterobacter*	Erysipelotrichaceae, *Clostridium*, *Holdemanella*
Mizutani *et al.* [39]	Ghana	80	–	16S rRNA	Norovirus, rotavirus	Yes	*Staphylococcus*, *Veillonella*, *Alloprevotella*, *Escherichia*	*Faecalibacterium*, *Subdoligranulum*
Dinleyici *et al.* [42]	Turkey	10	1 month	16S rRNA	Rotavirus	No	Gammaproteobacteria	*Bacteroides*, *Blautia*, *Ruminococcus*, *Faecalibacterium*

Asides *Escherichia* and *Streptococcus*, other bacteria have been found overabundant in diarrheal fecal microbiomes, even in the absence of global dysbiosis. Our research in Vietnam highlighted that these include *Fusobacterium mortiferum*, and several members of the human oral microbiota (*Granulicatella*, *Gemella*, *Actinomyces*, *Rothia*, *Fusobacterium nucleatum*, etc.) [15^•^], in line with other findings [14^•^,^19^,^27^]. The anaerobic *F. mortiferum* commonly colonizes the gastrointestinal tract (albeit in low abundance) of the Chinese, but not Western, population [28,29], and its proliferation has been recently noted in patients with colorectal polyps [30,31]. These suggest that *F. mortiferum* overabundance could be a general marker of gut dysbiosis, particularly in Asian populations. Computational analyses have suggested that oral bacteria could form a tight correlation network, as inferred from taxa co-abundance patterns, in the diarrheal gut microbiome [15^•^]. This indicates that they may co-exist in polymicrobial biofilms similar to those present in the oral cavity, but their significance in diarrhea diseases is not currently studied [32]. Microbial transit along the oral-gut axis occurs frequently in healthy individuals [33], and the ecologically barren landscape generated by diarrhea may be ideal for the transient colonization of these oral bacterial conglomerates.

Though overall dysbiosis patterns were not associated with different diarrheal etiologies [15^•^,^24^], there exists some nuanced variances. Bacteria-induced diarrhea was associated with an elevation of *Escherichia* [34], *Streptococcus* and oral bacteria [15^•^], while viral infections retained a higher abundance in *Bifidobacterium* [15^•^,^26^]. This may suggest that viral infections lead to a less severe reductions in anaerobic gut commensals [35,36], possibly because most viruses (rotavirus, norovirus) infect cells lining the small intestine, instead of the colon [37^•^,^38^]. In mouse model, rotavirus infection resulted in increased *Bacteroides* and *Akkermansia* populations (both with mucin-degrading capability) only in the ileal microbiome [37^•^], but evidence for the overgrowth of these two taxa in human rotavirus infections has been inconclusive [21,26,34,39]. On the other hand, *Giardia*-induced diarrhea was consistently linked to a decrease in Gammaproteobacteria and an enrichment of *Prevotella* [40]. Dysentery (mucoid/bloody diarrhea) is a severe form of infectious diarrhea with heightened gut inflammation, which requires antimicrobial treatments and longer hospitalization [10,41]. An overabundance of facultative anaerobes (*Escherichia*, *Streptococcus*, *Enterococcus*, etc.) has been reported in dysenteric diarrhea, which was coupled with a depletion in bacteria of known immunomodulatory effects (*Lactobacillus ruminis*, *Bifidobacterium pseudocatenulatum*) [14^•^,15^•^,^24^]. These findings indicate that bacterial infections and dysentery are usually accompanied with dysbiotic states diverging further from the healthy condition, which could be the effect of pathogen-triggered inflammation and/or frequent antimicrobial use.

## Post-diarrhea recovery phase

The gut microbiomes of patients recovering post-diarrhea diverge from those observed in the disease’s early phase and converge toward that in the healthy population. The recovery phase signals a gradual increase in taxonomic richness and diversity in the gut microbiome, but microbiome succession showed high temporal variability among the infected individuals [42]. By studying Bangladeshi patients infected with *V. cholerae* and enterotoxigenic *E. coli*, David *et al.* proposed a stepwise (mid-stage and late-stage) succession model for gut microbiome recovery [18^••^]. The expansion of *Escherichia*/*Streptococcus* eventually depletes the oxygen in the gut environment, leading to their population decline in the recovery phase. The mid-stage is specifically characterized by a sizable abundance of *Bacteroides* (occurring as early as day 7 since disease onset), while the late-stage harbors a greater abundance and diversity of *Prevotella* and SCFA-producing Firmicutes [18^••^,^19^,^24^,^27^]. Carbohydrate metabolism genes, mostly of the genus *Bacteroides*, were the most significantly enriched during the mid-stage, allowing these bacteria to flexibly extract energy from diet-derived and host-derived carbohydrates (plentiful in fiber and mucin, respectively) [18^••^,37^•^]. Notably, this chronological microbial assemblage resembles that of gut microbiome recovery post antimicrobial administration [43^••^]. Numerous studies have noted that following antimicrobial treatment, *Bacteroides* (or Bacteroidetes) flourish while Firmicutes and Actinobacteria diminish [44,45]. Similarly, iso-osmotic diarrhea induced a transient gut perturbation, with a significant *Bacteroides* bloom immediately post-washout [46]. *Bacteroides* species, such as *Bacteroides uniformis* and *Bacteroides thetaiotaomicron*, were identified as primary recovery-associated taxa due to their mucin-degrading capability [47,48]. By capitalizing on host-derived nutrients, *Bacteroides* becomes the keystone species for the colon’s ecological recovery. This subsequently initiates a complex network of cross-feeding to expedite the repopulation of other anaerobic and SCFA producing commensals (*Bifidobacterium*, *Roseburia, Faecalibacterium*, etc.), thus establishing a taxonomically and functionally diverse community [43^••^]. An outstanding question is whether the recovered microbiota returns to the pre-infection state in patients, and such data are limited due to the paucity of diarrhea cohort studies. Findings from a *Campylobacter* human challenge study showed that significant compositional differences still persisted when comparing the recovery and pre-infection microbiomes, with *Bacteroides* abundance during recovery attributed to antimicrobial use [49]. In contrast, the presence of the *Bacteroides*-enriched stage is less prominent in recovery from viral gastroenteritis [42], possibly owing to its less severe dysbiotic state and infrequent antimicrobial use.

## Further impact of diarrheal dysbiosis

Though diarrhea is mostly acute, repeated diarrhea episodes could exert lifelong consequences on a child health. Studies have long proposed that diarrhea and undernutrition amplify the effect of each other, which predisposes children to stunting, cognitive impairment, and glucose intolerance in adulthood [50]. Longitudinal microbiome tracking in Peruvian children demonstrated that increased diarrhea frequency substantially reduced the gut microbiome diversity and richness, and this effect was exacerbated in stunted children [16^••^]. Stunting was also associated with a slower rate in microbiome recovery, and the prolonged perturbation in turn reduced resilience to subsequent enteric infections, creating a vicious cycle of diarrhea and undernutrition. Stunted children in Africa were shown to have an overgrowth of oral bacteria in the small intestine and colon [51^•^]. This concurs with the evidence that macaques with growth faltering experienced taxonomic and functional alternations in their colonic microbiomes, with the preponderance of oral bacteria such as *Lactobacillus salivarius* and *Streptococcus* [52]. We speculate that repeated diarrhea increases the chance that translocated oral bacteria acclimatize to the perturbed gut environment, and their stable colonization might induce inflammation and alter the functionality of the microbiome. Indeed, colonic proliferation of oral bacteria is a known signature of colorectal cancer [53], and these bacteria (*F. nucleatum*, *Peptostreptococcus*) could potentiate tumorigenesis and enhance gut inflammation [54,55]. Asides from clinical diarrhea, asymptomatic carriage of enteropathogens also remodels the gut microbiome. Children infected with *Campylobacter*, norovirus or enteroaggregative *E. coli* had significant higher abundance of *Ruminococcus gnavus* [56], which has been robustly linked to Crohn disease and produced proinflammatory polysaccharides [57]. Similar to diarrhea, asymptomatic infection with *Campylobacter* was associated with stunting [56], highlighting the significance of dysbiosis outside the purging effect of diarrhea.

The expansion of Enterobacteriaceae during diarrhea’s early phase greatly increases its contact with the assault pathogens, thus heightening the likelihood of horizontal gene transfer. Experimental model has confirmed the ease of plasmid transfer from *Salmonella* to *E. coli*, owing to colitis-induced Enterobacteriaceae bloom [58]. Our study in Vietnam has identified that the same multidrug resistant plasmid was present in the commensal *E. coli* and pathogenic *Shigella sonnei*, both isolated from a single child with diarrhea [59^•^]. Moreover, the efficiency of plasmid transfer (from *S. sonnei* to *E. coli*) increased 10–40 folds when incubated with fluoroquinolone *in vitro*. This suggests that once pathogens enter settings with high enteric infection incidence and antimicrobial usage, the gut’s Enterobacteriaceae could act as an effective reservoir fostering the emergence of new multidrug resistance phenotype. This likely contributes to the rise of plasmid-mediated azithromycin resistance in *Shigella flexneri* 3a (once entered in the men-who-have-sex-with-men community) [60] and cephalosporin resistance in *S. sonnei* (once introduced into Vietnam) [61].

## Outlook

Despite the impressive reduction in diarrhea-related mortality globally, diarrhea endemicity and its incurred morbidity still remain a debilitating actor on child health. Gut dysbiosis following diarrhea is short-lived and reversible, but its negative effect is amplified in vulnerable populations. Outstanding questions remain on how dysbiosis mechanistically influences clinical resolution of diarrhea, and the contribution of dysbiosis on immunological functionality in long-term, given that gastroenteritis could increase the risks of ulcerative colitis, Guillain-Barré syndrome, and reactive arthritis [62]. Thorough understanding on the gut microbiome in undernourished children helped engineering a microbiota-directed complementary food, which successfully alleviated the microbiome immaturity and improved the health status in this target population [63^••^]. In light of negative results from recent probiotic trials for acute diarrhea [64,65], future research should exploit microbiome knowledge to design more optimal probiotics or interventions.

## Funding information

HCT is a Wellcome International Training Fellow (218726/Z/19/Z).

## Conflict of interest statement

Nothing declared.

## References and recommended reading

Papers of particular interest, published within the period of review, have been highlighted as:

• of special interest
•• of outstanding interest

## Declaration of Competing Interest

The authors report no declarations of interest.
